# Prediction of survival for patients with pemphigus vulgaris and pemphigus foliaceus: a retrospective cohort study

**DOI:** 10.1186/s13023-015-0263-4

**Published:** 2015-04-22

**Authors:** Adrian Baican, Roxana Chiorean, Daniel Corneliu Leucuta, Corina Baican, Sorina Danescu, Dorina Ciuce, Cassian Sitaru

**Affiliations:** Department of Dermatology, “Iuliu Hatieganu” University of Medicine and Pharmacy, Cluj-Napoca, Romania; Department of Dermatology, University Medical Center Freiburg, Hauptstrasse 7, 79104 Freiburg, Germany; Department of Medical Informatics and Biostatistics, “Iuliu Hatieganu” University of Medicine and Pharmacy, Cluj-Napoca, Romania; Centre for Biological Signalling Studies (BIOSS), University of Freiburg, Freiburg, Germany

**Keywords:** Risk factors, Pemphigus, Survival rate, Age factor, Cardiovascular disease, Anti-desmoglein 1 autoantibodies

## Abstract

**Background:**

Factors associated with survival in pemphigus have not yet been thoroughly addressed. Therefore, in the present study, risk factors for overall mortality in a large group of patients with pemphigus vulgaris and foliaceus were investigated.

**Methods:**

A retrospective hospital-based cohort study was carried out, between October 1998 and November 2012, in the Department of Dermatology of the University of Medicine and Pharmacy “Iuliu Hatieganu”, Cluj-Napoca, Romania. The investigated prognostic endpoint was the overall survival of the patients.

**Results:**

A total of 130 patients were studied (108 with pemphigus vulgaris and 22 with pemphigus foliaceus). In pemphigus vulgaris group, univariate analysis found a statistically significant association between the age of onset ≥ 65 years (p < 0.001), presence of coronary heart disease (p = 0.006), presence of cardiac arrhythmia (p = 0.004), level of anti-desmoglein1 autoantibodies ≥ 100 U/mL (p = 0.047) at diagnosis and the survival of the patients. An age-adjusted analysis showed significant results for coronary heart disease. Multivariate analysis identified the age of onset ≥ 65 years and the presence of coronary heart disease at diagnosis as independent risk factors associated with overall mortality. In patients with pemphigus foliaceus, age of onset ≥ 65 years (p = 0.021) was associated with poor survival.

**Conclusions:**

In addition to common prognostic factors, including older age and cardiovascular comorbidities, level of autoantibodies was found to be a disease-specific factor associated with overall mortality in pemphigus vulgaris. The newly identified factors have major implications for the stratification of patients and should greatly facilitate further epidemiological studies in pemphigus. In addition, they provide useful information for the design of personalized therapeutic plans in the clinical setting.

## Background

Pemphigus encompasses a group of autoimmune blistering skin diseases, characterized by autoreactive T and B lymphocytes, targeting antigens of the intercellular junctions of keratinocytes [1]. Two major forms of pemphigus, pemphigus vulgaris (PV; ORPHA704) and pemphigus foliaceus (PF; ORPHA79481), show specific clinical, histological and immunopathological findings [2]. If not treated, pemphigus vulgaris is often a fatal disease. Despite the fact that prognosis of the condition has dramatically improved after the advent of glucocorticoids and immunosuppresive therapy, recent population-based studies documented an increased risk of death in patients with pemphigus [3,4].

While several studies have been undertaken to evaluate the risk factors for disease course, relapses and remissions in patients with pemphigus [5-8], few investigations evaluated the risk factors for overall mortality in patients with PV [9-12] and none for PF as a separate entity.

Previous studies provided fragmented information suggesting that an older age of onset [3,10,11,13], concomitant involvement of the skin and mucosal surface at early disease presentation [9,14,15], delayed initiation of corticosteroid treatment [9] and the therapeutic combination of corticosteroids plus azathioprine, vs corticosteroids alone [11] are possible risk factors for overall mortality in patients with pemphigus. Further putative risk factors, including comorbidities and pathogenetically-relevant immunopathological features have not yet been assessed sistematically. Therefore, in a hospital-based retrospective approach, we aimed to identify possible risk factors for overall mortality in a characterised group of patients with PV and PF*.*

## Methods

### Design overview

This retrospective cohort study was performed in the Department of Dermatology at the University of Medicine and Pharmacy “Iuliu Hatieganu”, from Cluj-Napoca, Romania. Patients diagnosed between October 1998 and November 2012 were included. The study was approved by the local Ethic Committee and performed in accordance with the Declaration of Helsinki.

### Patients

The study group consisted of hospitalized or ambulatory patients, which were included in clinical files and electronic data bases. Epidemiological, clinical and immunological data were collected from patients' medical records. Death records data were obtained from the National Population Register. Missing data from patients’ files were obtained from the patients themselves by the use of telephone interviews. An informed consent was signed by all patients.

Inclusion criteria for patients with PV were: 1) presence of skin bullae and/or erosions on mucous membranes, 2) intraepidermal acantholysis on histopathological examination of skin and/or mucosa biopsies, 3) intraepidermal IgG and/or C3 deposits by direct immunofluorescence and presence of anti-desmoglein 3 +/− anti-desmoglein 1 autoantibodies, measured by ELISA method. Inclusion criteria for patients with PF were: 1) presence of skin bullae, 2) lack of mucosal lesions, 3) intraepidermal acantholysis on histopathological examination of skin biopsies, 4) intraepidermal IgG and/or C3 deposits by direct immunofluorescence and presence of anti-desmoglein 1 autoantibodies, with lack of anti-desmoglein 3 autoantibodies, measured by enzyme-linked immunosorbent assay (ELISA) method. Patients diagnosed with other subtypes than PV or PF were not included in our study.

The collected epidemiological variables were the age of onset, gender, ethnicity, alcohol consumption and smoking status. The clinical variables were the subtype of disease (PV/PF), subtype of pemphigus vulgaris (cutaneous, mucosal, mucocutaneous), associated comorbidities at diagnosis, value of erythrocyte sedimentation rate (ESR) (mm/h) at diagnosis, initial dose of corticosteroids (mg/kg/day) and follow-up time. The immunopathological features recorded were the direct immunofluorescence microscopy (DIF) findings, titres of anti-desmoglein1 (Dsg1) and anti-desmoglein3 (Dsg3) autoantibodies.

### Collection of immunopathological data

DIF was performed in all patients. In order to determine levels of specific anti-Dsg1 and anti-Dsg3 autoantibodies at diagnosis, commercially available ELISA kits were used (MBL laboratories, Nagoya, Japan). The cut-off value for positive levels of anti-desmoglein1 and anti-desmoglein3 autoantibodies was taken as ≥ 20 U/mL. To use standardized conditions, reduce bias and pre-analytical sources of error, sera from newly diagnosed patients were frozen after collection. Subsequently, ELISA analyses were performed at regular intervals.

### Endpoints

The overall survival (OS) rate, representing the period (months) from diagnosis to death, was chosen as endpoint. Overall survival was assessed after a follow-up of minimum 12 months.

For the survival analysis, the cut off values used for numeric continuous variables were similar to those found in the literature. Age was assessed both as a continuous and dichotomous variable. Since information on age as a risk factor for overall mortality in pemphigus is largely lacking, in line with the World Health Organisation definition of an “elderly” or “older” person [16] we used the cut-off value of 65 years. A level of ESR > 30 mm/h has been previously described as risk factor for death among patients with bullous pemphigoid [17]. Therefore, we have chosen it as cut-off value.

A previous categorization of levels of anti-desmoglein autoantibodies has been made by Saha et al. [8], who divided the values in three groups, as follows: 0–29 (negative), 30–100 (low and medium levels), >100 (high levels). For the levels of anti-desmoglein 1 and anti-desmoglein 3 autoantibodies, we have taken as cut-off value the level of 100 U/ml, in order to check if there was a survival difference between patients with high levels and patients with negative, low and medium levels, as previously described.

The cut-off value for the corticosteroid dose was 1 mg/kg/day.

### Statistical analysis

Association between potential risk factors (epidemiological, clinical and immunopathological variables defined above) was assessed using the log-rank test and the Kaplan Meier curves. The hazard ratios (HR) were computed for all potential risk factors after adjusting for the effect of age as a continuous and dichotomous variable using a Cox proportional hazard model. Subsequently, a multivariate Cox regression analysis was performed, using Akaike information criterion for stepwise backward/forward model selection. Hazard ratios are presented with 95% confidence intervals. The Mann Whitney U test, medians and interquartile range were used for non‐normally distributed variables. For all tests, a two-tailed p-value < 0.05 was considered statistically significant. Statistical analysis was performed using the R software environment for statistical computing and graphics [18].

## Results

**Figure 1:**
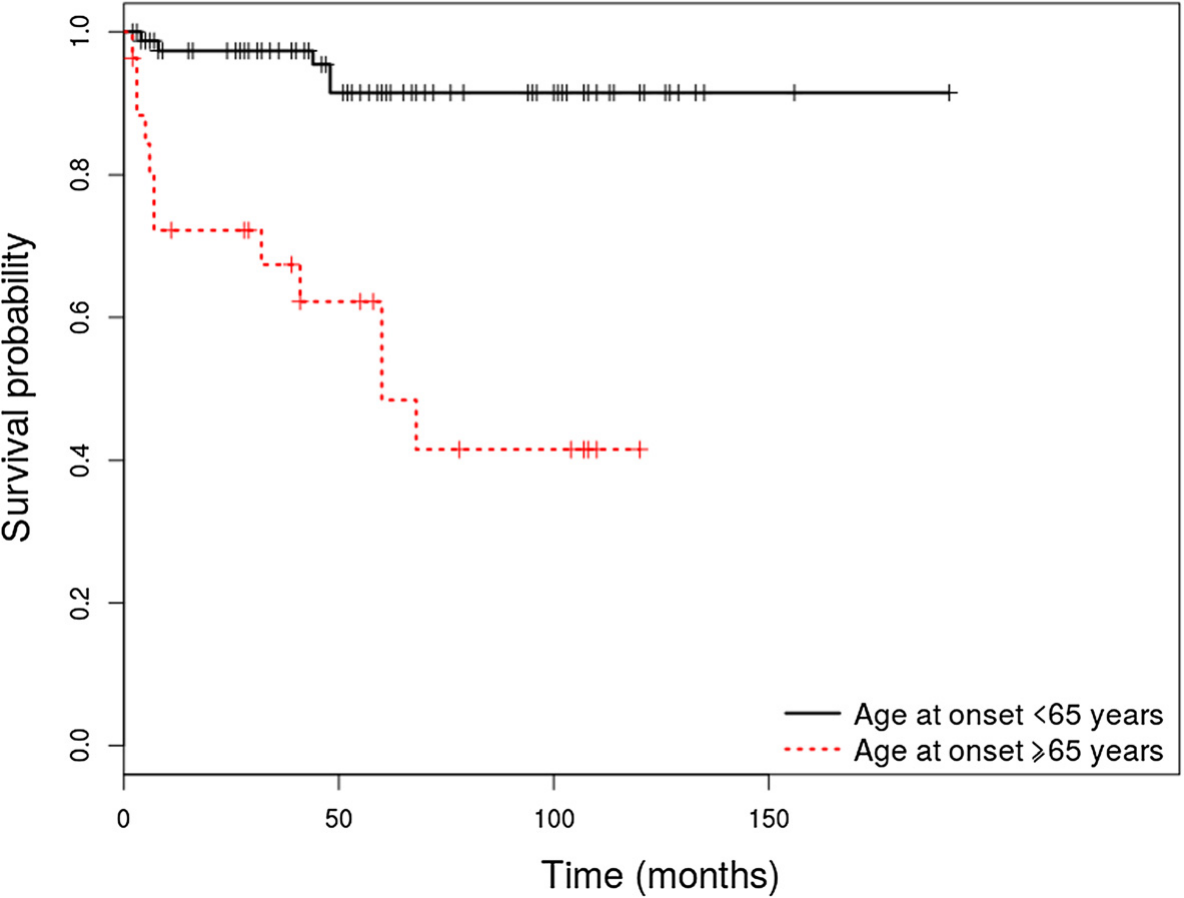
Kaplan-Meier analysis of survival in PV patients with an age of onset ≥ 65 years vs. patients with an age of onset <65 years.

**Figure 2:**
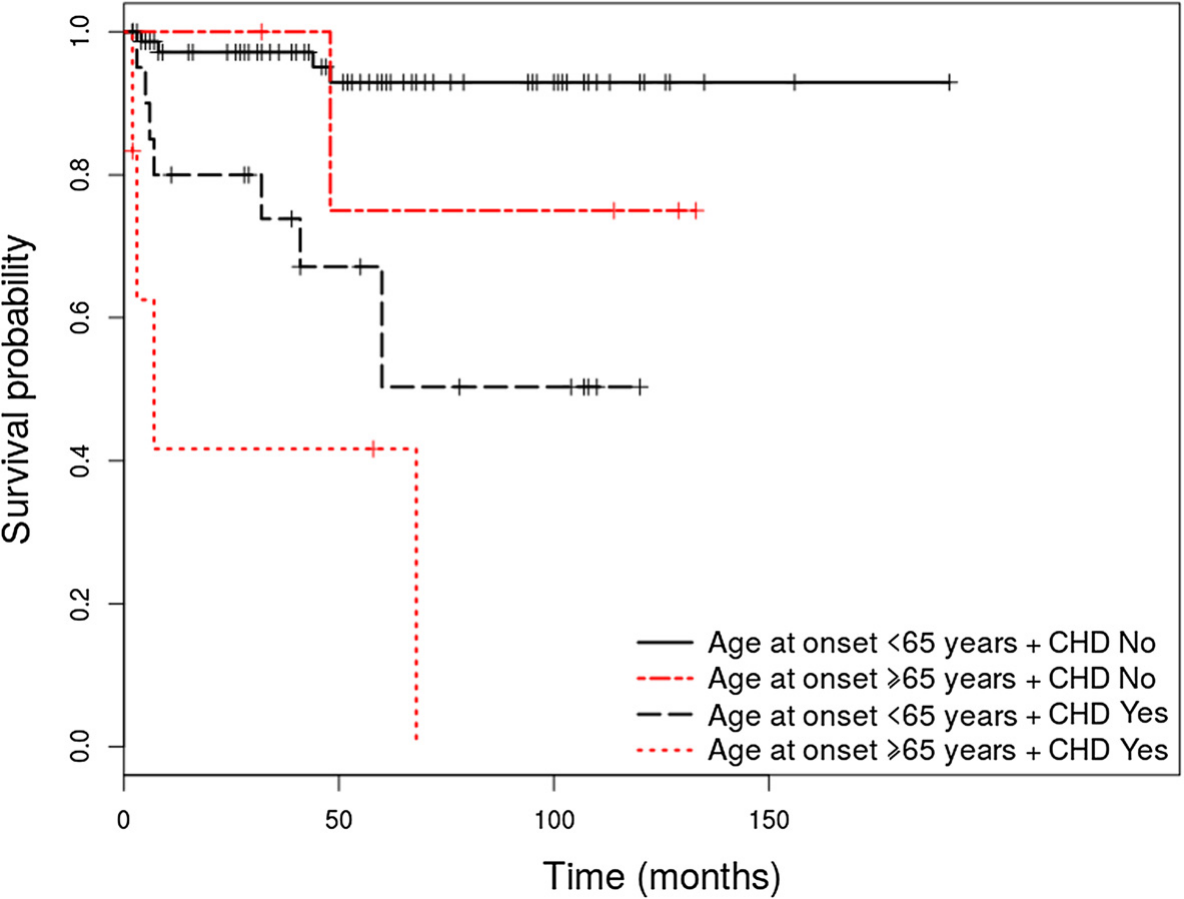
Kaplan-Meier analysis of survival in PV patients with coronary heart disease (CHD) vs. patients without coronary heart disease (CHD) at diagnosis, stratified by age.

**Figure 3:**
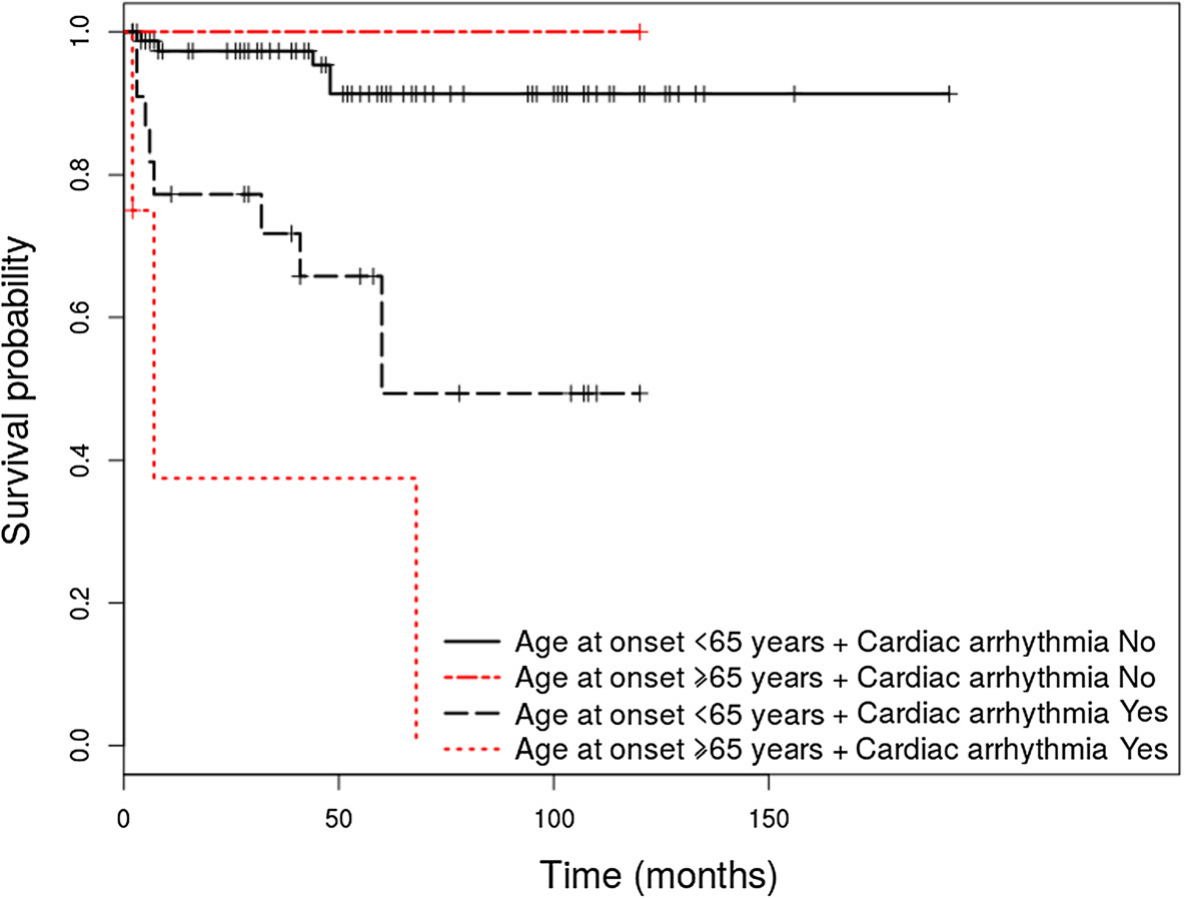
Kaplan-Meier analysis of survival in PV patients with cardiac arrhythmia vs. patients without cardiac arrhythmia at diagnosis, stratified by age.

**Figure 4:**
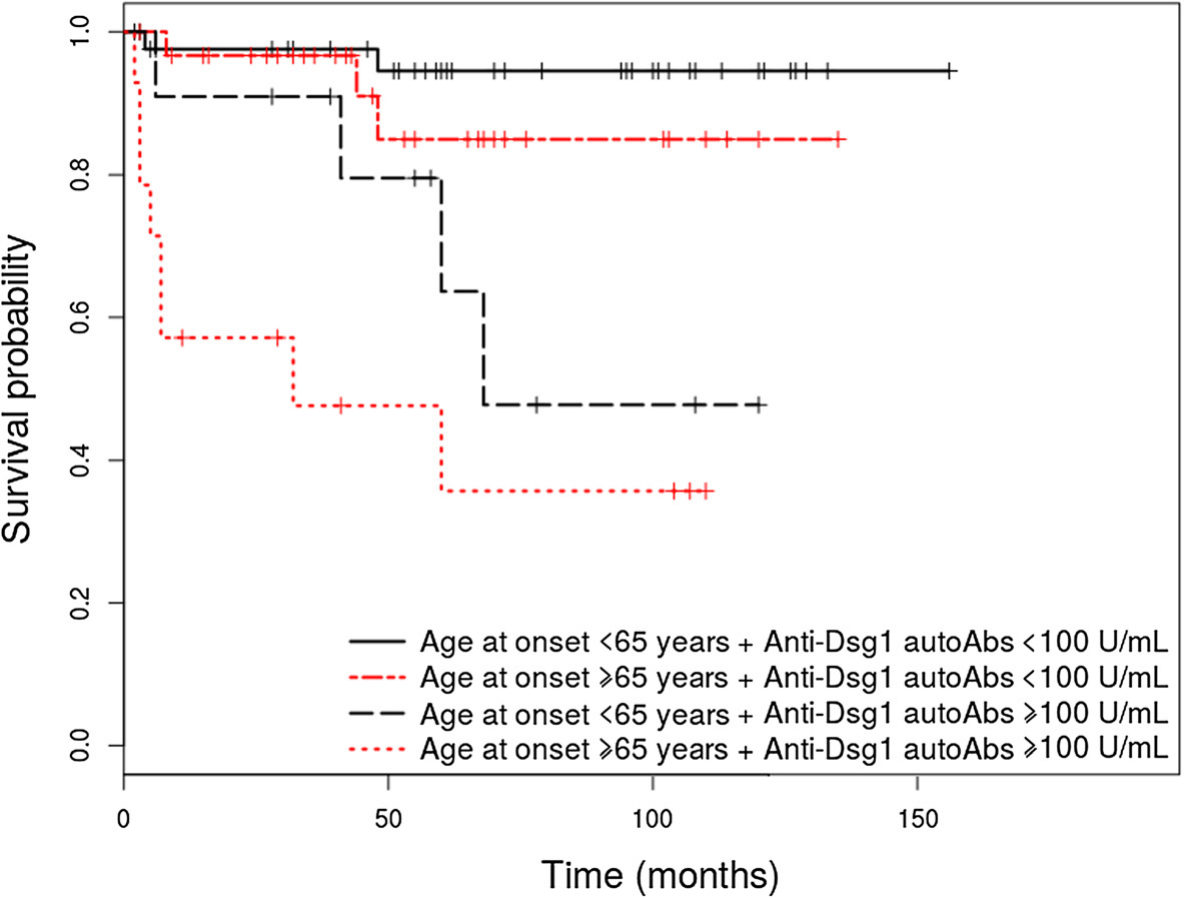
Kaplan-Meier analysis of survival in PV patients with level of anti-Dsg1 autoantibodies ≥ 100 U/mL vs. patients with level of anti-Dsg1 autoantibodies <100 U/mL at diagnosis, stratified by age.

Out of the 130 patients enrolled in the study, 108 (83.1%) were diagnosed with PV and 22 (16.9%) with PF. The HRs adjusted for age of onset as continuous variable are shown in Table 1 for PV patients and Table 2 for PF patients. Survival differences between groups at 1, 3, 5 and 10 years are shown in Table 3 for patients with PV and Table 4 for patients with PF. Only relevant Kaplan Meier curves have been shown (Figures 1, 2, 3 and 4).

**Table 1:** **Age of onset in years, adjusted HR analysis for overall survival rates in patients with pemphigus vulgaris**

**Characteristic**	**No (%)**	**Deaths**	**HR (95% CI)**	**p-value**
		**No (%)**		
Age of onset ≥ 65 years	27 (25.0%)	12 (70.6%)	9.37 (95%CI 3.3 – 26.65)	<0.001
Age of onset < 65 years	81 (75.0%)	5 (29.4%)		
Gender (M)	46 (42.6%)	6 (35.3%)	0.73 (0.26 – 2.09)	0.561
Gender (F)	62 (57.4%)	11 (64.7%)		
Ethnicity (Romanian)	100 (92.6%)	16 (94.1%)	0.57 (0.07 – 4.12)	0.588
Ethnicity (Roma)	8 (7.4%)	1 (5.9%)		
Alcohol consumption (Yes)	20 (18.5%)	3 (17.7%)	1.52 (0.42 – 5.51)	0.524
Alcohol consumption (No)	88 (81.5%)	14 (82.3%)		
Smoking (Yes)	29 (26.9%)	2 (11.8%)	1.36 (0.29 – 6.33)	0.698
Smoking (No)	79 (73.1%)	15 (88.2%)		
Subtype of PV				
Mucocutaneous	80 (74.1%)	14 (82.3%)	2.60 (0.34 – 19.93)	0.357
Cutaneous	13 (12.0%)	1 (5.9%)		
Mucous	15 (13.9%)	2 (11.8%)	1.72 (0.16 – 18.97)	0.659
High blood pressure (Yes)	28 (25.9%)	6 (35.3%)	0.95 (0.35 – 2.60)	0.928
High blood pressure (No)	80 (74.1%)	11 (64.7%)		
Coronary heart disease (Yes)	11 (10.2%)	5 (29.4%)	3.04 (1.07 – 8.67)	0.037
Coronary heart disease (No)	97 (89.8%)	12 (70.6%)		
Heart failure (Yes)	4 (3.7%)	1 (5.9%)	0.63 (0.08 – 4.76)	0.651
Heart failure (No)	104 (96.3%)	16 (94.1%)		
Cardiac arrhythmia (Yes)	5 (4.6%)	3 (17.7%)	2.70 (0.75 – 9.59)	0.125
Cardiac arrhythmia (No)	103 (95.4%)	14 (82.3%)		
Diabetes mellitus (Yes)	8 (7.4%)	2 (11.8%)	0.91 (0.21 – 3.99)	0.898
Diabetes mellitus (No)	100 (92.6%)	15 (88.2%)		
Stroke (Yes)	5 (4.6%)	1 (5.9%)	1.08 (0.14 – 8.22)	0.944
Stroke (No)	103 (95.4%)	16 (94.1%)		
Depression (Yes)	5 (4.6%)	1 (5.9%)	2.09 (0.27 – 16.22)	0.480
Depression (No)	103 (95.4%)	16 (94.1%)		
Dental problems (Yes)	57 (52.8%)	7 (41.2%)	0.85 (0.32 – 2.28)	0.750
Dental problems (No)	51 (47.2%)	10 (58.8%)		
ESR ≥ 30 mm/hour	26 (42.6%)	9 (75.0%)	1.51 (0.37 – 6.20)	0.570
ESR < 30 mm/hour	35 (57.4%)	3 (25.0%)		
Steroids ≥ 1 mg/kg/day	15 (15.0%)	2 (14.3%)	0.88 (0.19 – 4.05)	0.868
Steroids < 1 mg/kg/day	85 (85.0%)	12 (85.7%)		
Anti-Dsg1 autoAbs ≥ 100 U/mL	45 (45.5%)	11 (64.7%)	1.98 (0.71 – 5.53)	0.195
Anti-Dsg1 autoAbs < 100 U/mL	54 (54.5%)	6 (35.3%)		
Anti-Dsg3 autoAbs ≥ 100 U/mL	71 (71.7%)	11 (64.7%)	0.60 (0.22 – 1.63)	0.314
Anti-Dsg3 autoAbs <100 U/mL	28 (28.3%)	6 (35.3%)

**Table 2:** **Age of onset in years, adjusted HR analysis for overall survival rates in patients with pemphigus foliaceus**

**Characteristic**	**No (%)**	**Deaths**	**HR (95% CI)**	**p-value**
		**No (%)**		
Age of onset ≥65 years	7 (31.8%)	4 (80.0%)	13.48 (1.49 – 121.94)	0.021
Age of onset <65 years	15 (68.2%)	1 (20.0%)		
Gender (M)	4 (18.2%)	0 (0.0%)	NA	NA
Gender (F)	18 (81.8%)	5 (100.0%)		
Alcohol (Yes)	2 (9.1%)	0 (0.0%)	NA	NA
Alcohol (No)	20 (90.9%)	5 (100.0%)		
Smoking (Yes)	9 (40.9%)	0 (0.0%)	NA	NA
Smoking (No)	13 (59.1%)	5 (100.0%)		
High blood pressure (Yes)	6 (27.3%)	2 (40.0%)	0.58 (0.09 – 3.80)	0.570
High blood pressure (No)	16 (72.7%)	3 (60.0%)		
Coronary heart disease (Yes)	3 (13.6%)	1 (20.0%)	3.07 (0.26 – 36.36)	0.375
Coronary heart disease (No)	19 (86.4%)	4 (80.0%)		
Diabetes mellitus (Yes)	2 (9.1%)	1 (20.0%)	7.79 (0.54 – 111.18)	0.130
Diabetes mellitus (No)	20 (90.9%)	4 (80.0%)		
Dental problems (Yes)	12 (54.5%)	2 (40.0%)	1.77 (0.24 – 13.24)	0.579
Dental problems (No)	10 (45.5%)	3 (60.0%)		
ESR ≥ 30 mm/hour	7 (31.8%)	2 (40.0%)	NA	NA
ESR < 30 mm/hour	5 (22.7%)	0 (0.0%)		
Steroids ≥ 1mg/kg/day	4 (18.2%)	1 (20.0%)	NA	NA
Steroids < 1 mg/kg/day	12 (54.5%)	2 (40.0%)		
Anti-Dsg1 autoAbs ≥ 100 U/mL	12 (54.5%)	4 (80.0%)	2.59 (0.28 – 24.13)	0.404
Anti-Dsg1 autoAbs < 100 U/mL	10 (45.5%)	1 (20.0%)

**Table 3:** **Differences between survival rates at 1, 3, 5 and 10 years, in patients with pemphigus vulgaris**

**Characteristic**	**Survival rate (%)**	**3**	**5**	**10**	**p-value**
	**Follow up (years) 1**				
Age of onset ≥ 65 years	72.2%	67.4%	48.4%	41.5%	< 0.001
Age of onset < 65 years	97.3%	97.3%	91.4%	91.4%	
*Survival difference (%)*	*25.1%*	*29.9%*	*43.0%*	*49.9%*	
Gender (M)	88.0%	88.0%	83.6%	83.6%	0.611
Gender (F)	93.3%	91.3%	78.7%	75.1%	
*Survival difference (%)*	5.3%	3.3%	4.9%	8.5%	
Ethnicity (Romanian)	90.6%	89.3%	81.1%	79.0%	0.816
Ethnicity (Roma)	100.0%	100.0%	75.0%	75.0%	
*Survival difference (%)*	9.4%	10.7%	6.1%	4.0%	
Alcohol consumption (Yes)	83.3%	83.3%	83.3%	83.3%	0.997
Alcohol consumption (No)	92.8%	91.4%	80.1%	77.5%	
*Survival difference (%)*	9.5%	8.1%	3.2%	5.8%	
Smoking (Yes)	92.1%	92.1%	92.1%	92.1%	0.299
Smoking (No)	90.9%	89.4%	78.1%	75.6%	
*Survival difference (%)*	1.2%	2.7%	14.0%	16.5%	
Subtype of PV					
Mucocutaneous	90.8%	89.2%	76.8%	76.8%	0.692
Cutaneous	92.3%	92.3%	92.3%	92.3%	
Mucous	92.9%	92.9%	92.9%	79.6%	
High blood pressure (Yes)	88.5%	83.6%	77.2 %	70.7 %	0.303
High blood pressure (No)	92.0%	92.0%	81.8%	81.8%	
*Survival difference (%)*	3.5%	8.4%	4.6%	11.1%	
Coronary heart disease (Yes)	70.7%	70.7%	58.9%	44.2%	0.003
Coronary heart disease (No)	93.4%	92.0%	83.1%	83.1%	
*Survival difference (%)*	22.7%	21.3%	24.2%	38.9%	
Heart failure (Yes)	NA	100.0%	100.0%	0.0%	0.697
Heart failure (No)	90.8%	89.5%	79.9%	79.9%	
*Survival difference (%)*	NA	10.5%	20.1%	79.9%	
Cardiac arrhythmia (Yes)	53.3%	53.3%	53.3%	26.7%	0.001
Cardiac arrhythmia (No)	92.8%	91.5%	81.7%	81.7%	
*Survival difference (%)*	39.5%	38.2%	28.4%	55.0%	
Diabetes mellitus (Yes)	87.5%	87.5%	65.6%	65.6%	
Diabetes mellitus (No)	91.5%	90.2%	81.8%	79.6%	
*Survival difference (%)*	4.0%	2.7%	16.2%	14.0%	0.438
Stroke (Yes)	80.0%	80.0%	80.0%	80.0%	0.749
Stroke (No)	91.7%	90.5%	80.6%	78.4%	
*Survival difference (%)*	11.7%	10.5%	0.6%	1.6%	
Depression (Yes)	80.0%	80.0%	80.0 %	80.0%	0.629
Depression (No)	91.7%	90.4%	80.8%	78.7%	
*Survival difference (%)*	11.7%	10.4%	0.8%	1.3%	
Dental problems (Yes)	90.5%	88.0%	84.8%	84.8%	0.508
Dental problems (No)	91.9%	91.9%	77.2%	73.6%	
*Survival difference (%)*	1.4%	3.9%	7.6%	11.2%	
ESR ≥ 30 mm/hour	84.6%	80.4%	66.4%	60.9%	0.039
ESR < 30 mm/hour	93.6%	93.6%	88.9%	88.9%	
*Survival difference (%)*	9.0%	13.2%	22.5%	28.0%	
Steroids ≥ 1 mg/kg/day	93.3%	93.3%	84.9%	84.8%	0.726
Steroids < 1 mg/kg/day	93.6%	92.0%	81.0%	78.1%	
*Survival difference (%)*	0.3%	1.3%	3.9%	6.7%	
Anti-Dsg1 autoAbs ≥ 100 U/mL	84.2 %	81.3%	68.8%	68.8%	0.039
Anti-Dsg1 autoAbs < 100 U/mL	96.1 %	96.1%	88.3%	84.9%	
*Survival difference (%)*	11.9 %	14.8%	19.5 %	16.1%	
Anti-Dsg3 auto Abs ≥ 100 U/mL	91.3%	89.5%	80.4%	80.4%	0.506
Anti-Dsg3 auto Abs < 100 U/mL	89.3%	89.3%	78.8%	71.6%	
*Survival difference (%)*	2.0%	0.2%	1.6%	8.8%

**Table 4:** **Differences between survival rates at 1, 3, 5 and 10 years, in patients with pemphigus foliaceus**

**Characteristic**	**Survival rate (%) ** **Follow up (years)** **1**	**3**	**5**	**10**	**p-value**
Age of onset ≥ 65 years	68.6%	34.3%	34.3%	34.3%	0.003
Age of onset < 65 years	NA	93.3%	93.3%	93.3%	
*Survival difference (%)*	NA	59.0%	59.0%	59.0%	
Gender (M)	NA	NA	100.0%	100.0%	NA
Gender (F)	88.5%	70.8%	70.8%	70.8%	
*Survival difference (%)*	NA	NA	29.2%	29.2%	
Alcohol (Yes)	NA	NA	NA	100.0%	NA
Alcohol (No)	89.7%	73.9%	73.9%	73.9%	
*Survival difference (%)*	NA	NA	NA	26.1%	
Smoking (Yes)	NA	100.0%	100.0%	100.0%	NA
Smoking (No)	83.9%	58.7%	58.7%	58.7%	
*Survival difference (%)*	NA	41.3%	41.3%	41.3%	
High blood pressure (Yes)	83.3%	66.7%	66.7%	66.7%	0.518
High blood pressure (No)	93.3%	80.0%	80.0%	80.0%	
*Survival difference (%)*	10.0%	13.3%	13.3%	13.3%	
Coronary heart disease (Yes)	66.7%	66.7%	66.7%	66.7%	0.545
Coronary heart disease (No)	94.4%	77.8%	77.8%	77.8%	
*Survival difference (%)*	27.7%	11.1%	11.1%	11.1%	
Diabetes mellitus (Yes)	50.0%	50.0%	50.0 %	50.0%	0.197
Diabetes mellitus (No)	94.7%	79.0%	79.0%	79.0%	
*Survival difference (%)*	44.7%	29.0%	29.0%	29.0%	
Dental problems (Yes)	82.5%	82.5%	82.5%	82.5%	0.623
Dental problems (No)	NA	70.0%	70.0%	70.0%	
*Survival difference (%)*	NA	12.5%	12.5%	12.5%	
ESR ≥ 30 mm/hour	NA	71.4%	71.4%	71.4%	NA
ESR < 30 mm/hour	100.0%	100.0%	100.0%	100.0%	
*Survival difference (%)*	NA	28.6%	28.6%	28.6%	
Steroids ≥ 1 mg/kg/day	NA	75.0%	75.0%	75.0%	0.695
Steroids < 1 mg/kg/day	100.0%	81.8%	81.8%	81.8%	
*Survival difference (%)*	NA	6.8%	6.8%	6.8%	
Anti-Dsg1 autoAbs ≥ 100 U/mL	83.3%	66.7%	66.7%	66.7%	0.313
Anti-Dsg1 autoAbs < 100 U/mL	NA	87.5%	87.5%	87.5%	
*Survival difference (%)*	83.3%	20.8%	20.8%	20.8%

### Outcome data

Among patients with PV, a total of 17 died (11 females, 6 males), 9 of them within the first year after diagnosis. The median overall survival among the patients who died was 8 months [2–68]. The median duration of follow up among the patients who were alive was 58 months [2–192]. Among patients with PF, 5 died, 2 of them within the first year after diagnosis. All of them were females. The median overall survival among the patients who died was 17 months [5-36]. The median duration of follow up among the patients who were alive was 68 months [6–126]. In the pemphigus vulgaris group, the cause of death was retrievable in 7 patients, out of which 4 showed an “early death”, within the first year after diagnosis. A number of 5 patients died of “pemphigus-related” causes, out of which 3 within the first year. One patient refused to pursue the medication and died of malnutrition, 6 months after diagnosis. One patient developed an extended form of pemphigus and died of MRSA sepsis, 7 months after diagnosis. Three patients developed an aggressive relapse and died due to the complications induced by corticosteroid therapy (one with gastrointestinal bleeding, one with pulmonary embolism and one with cardiac insufficiency), one at 2 months after diagnosis (gastrointestinal bleeding). Two patients died of ''non-pemphigus related'' causes (one of acute myocardial infarction, 3 months after diagnosis and one of stroke).

In the pemphigus foliaceus group, the cause of death was available in 2 patients. One patient died of ''pemphigus-related causes'', within one year after diagnosis. She developed a relapse and refused to continue the treatment. Additionally, she had a type 2 diabetes mellitus and renal-failure. One patient died of ''non-pemphigus related'' cause (i.e., colorectal carcinoma) 3 years after diagnosis.

The median interval from onset to diagnosis in the pemphigus vulgaris group was 3 months, ranging between 0 and 48 months. The median interval from onset to diagnosis in the pemphigus foliaceus group was 5 months, ranging between 1 and 36 months.

The interval between onset and diagnosis in the PV group of patients who died within the first year after diagnosis varied between 0 and 7 months. Seven of these patients had an age > 65 years and 2 patients had an age < 65 years. In the pemphigus foliaceus group, two patients died within the first year after diagnosis. Both had an age > 65 years. They were diagnosed 1, respectively 2 months after the onset.

### Influence of epidemiological characteristics on the overall survival rate

#### *An age of onset* ≥ *65 years is associated with a higher risk of overall mortality in patients with pemphigus vulgaris and foliaceus*

The median age of onset was 50 years [15–83] in the PV group and 53 [26–87] in the PF group. PV patients with an age of onset ≥ 65 years showed a higher risk for overall mortality, compared to patients with an age of onset < 65 years (HR = 9.37 (95% CI, 3.3-26.65), p < 0.001). Age of onset taken as a continuous variable also represented a risk factor for overall mortality (HR age = 1.12 (95% CI, 1.06 – 1.18), p < 0.001). PV patients with an age of onset ≥ 65 years also demonstrated a lower OS rate at 1, 3, 5 and 10 years (p < 0.001). PF patients with an age of onset ≥ 65 years showed a higher risk for overall mortality, compared to patients with an age of onset < 65 years (HR = 13.48 (95% CI, 1.49 –121.94), p = 0.021). In this subgroup, age taken as a continuous variable also represented a risk factor for overall mortality (HR age = 1.09 (95% CI,1.01 – 1.16), p = 0.025). These patients also demonstrated a lower OS rate at 3, 5 and 10 years (p = 0.003).

Female to male ratio was 1.34:1 among patients with PV and 3:1 among those with PF. The main ethnicity in our studied population was Romanian (92.6% of patients with PV and 100% of patients with PF). The second ethnicity was Roma (7.4% of patients with PV). Gender and ethnicity were not found to be related to overall mortality and did not influence the OS in the PV and PF groups (p > 0.05).

We could not find alcohol intake and smoking status as statistically significant risk factors for overall mortality in patients with PV and PF (p > 0.05). Still, smokers with both PV and PF had a higher OS rate, compared to non-smokers (p > 0.05).

### Influence of clinical characteristics on the overall survival rate

#### Presence of coronary heart disease and cardiac arrhythmia at diagnosis is associated with a higher risk of overall mortality in patients with pemphigus vulgaris

Subtype of pemphigus (PV vs. PF) was not found to be related to overall mortality (p = 0.701). Although patients with PV showed a slightly higher OS survival rate at 1, 3, 5 and 10 years, compared to patients with PF, the results were not statistically significant (p = 0.559).

Of the 108 patients with PV, 80 (74.1%) presented mucocutaneous involvement, 13 (12.0%) presented only cutaneous involvement, while 15 (13.9%) presented only mucous involvement. The subtype of PV (mucocutaneos, cutaneous, mucous) was not found to be related to overall mortality (p > 0.05). Patients with mucocutaneous subtype showed lower OS rates, at 1, 3, 5 and 10 years compared to patients with cutaneous and mucous subtype (p > 0.05).

The evaluated comorbidity profile in patients with PV consisted of the following nosological entities: high blood pressure, coronary heart disease, heart failure, cardiac arrhythmia, diabetes mellitus, stroke, depression and dental problems. None of the patients with PF associated heart failure, cardiac arrhythmia, stroke and depression as comorbidities. Thus, these variables have not been assessed as risk factors in patients with PF. Presence of these comorbidities was assessed at diagnosis, in a cross-sectional manner (presence or absence of the comorbidity at diagnosis). Patients have been diagnosed with coronary heart disease by a cardiologist or general practitioner, before the diagnosis of pemphigus has been made. Coronary heart disease was defined according to the criteria included in the guidelines of the European Society of Cardiology [19].

In the PV group, association of coronary heart disease (HR = 4.27 (95% CI, 1.5- 12.13), p = 0.006) and cardiac arrhythmia (HR = 6.19 (95% CI, 1.77-21.64), p = 0.004) at diagnosis represented statistically significant risk factors for overall mortality in the initial univariate analysis. Adjusting by age, revealed coronary heart disease as a statistically significant risk factor for overall mortality (HR = 3.04 (95% CI, 1.07 – 8.67), p = 0.037 for age adjusted as continuous variable and HR = 2.96 (95% CI,1.03 – 8.54), p = 0.044 for age adjusted as dichotomous variable), while cardiac arrhythmia did not represent a statistically significant risk factor for overall mortality (HR = 2.70 (0.75 – 9.59), p = 0.125, for age adjusted as continuous variable and HR = 2.71 (0.75 – 9.80), p = 0.129, for age adjusted as dichotomous variable). Patients who associated these comorbidities at diagnosis have also shown lower OS rates at 1, 3, 5 and 10 years, compared to patients who did not associate coronary disease or cardiac arrhythmia at diagnosis (p = 0.003 for coronary heart disease and p = 0.001 for cardiac arrhythmia). Although coronary disease was not found to be related to overall mortality in patients with PF (p = 0.375), it has been associated with a decreased OS rate at 1, 3, 5 and 10 years (p = 0.545).

High blood pressure, diabetes mellitus and dental problems were not found to be related to overall mortality in patients with PV and PF (p > 0.05). Patients showing high blood pressure and diabetes mellitus displayed a decreased OS rate at 1, 3, 5 and 10 years, compared to patients with PV or PF who did not associate these comorbidities at diagnosis (p > 0.05).

Presence of heart failure, stroke and depression at diagnosis were not found as additional risk factors for overall mortality in patients with PV. A history of stroke and depression was associated with a decreased OS rate at 1, 3 and 5 years, in patients with PV (p > 0.05).

The median ESR value was 27 mm/h [4–104] in pemphigus vulgaris group and 35 mm/h [4–105] in pemphigus foliaceus group. PV patients with an ESR ≥ 30 mm/h showed a statistically significant decreased OS rate at 1, 3, 5 and 10 years, compared to patients with an ESR < 30 mm/h (p = 0.039). An ESR ≥ 30 mm/h did not represent a risk factor for overall mortality in patients with PV (p > 0.05).

Regarding the administrated treatment in the PV group, 108 patients received corticosteroids. Out of this group, a number of 31 patients received corticosteroids alone. 53 patients received corticosteroids in combination with azathioprine, 9 patients received corticosteroids in combination with cyclophosphamide, while 12 patients received corticosteroids in combination with azathioprine and cyclophosphamide. One patient received corticosteroids in combination with methotrexate. Two patients additionally received intravenous immunoglobulins. One patient received dapsone alone.

Out of a total number of 22 patients with PF, 12 received corticosteroids alone, while 9 received corticosteroids plus azathioprine. One patient received only topical steroids.

The mean initial dose of corticosteroids was 0.7 mg/kg/day [0–2] in patients with PV and 0.6 mg/kg/day [0–1.5] in patients with PF. An initial dose of corticosteroids ≥1 mg/kg was not found to be related to overall mortality in patients with PV or PF (p > 0.05). Also, no differences between survival at 1, 3, 5 and 10 years have been observed between patients with an initial dose of steroids ≥ 1 mg/kg/day and patients with an initial dose of steroids < 1 mg/kg/day (p > 0.05).

### Influence of immunopathological characteristics on the overall survival rate

#### *A level of anti-desmoglein 1 autoantibodies* ≥*100 U/mL at diagnosis associates with a higher risk of overall mortality in patients with pemphigus vulgaris*

Values of anti-Dsg1 and anti-Dsg3 autoantibodies were measured in 99 patients with PV and 22 patients with PF. In the PV group, anti-Dsg1 autoantibodies were positive in 57 patients (71.2%) with mucocutaneous type, 8 patients (61.5%) with cutaneous type and 3 patients (20.0%) with mucous type. Anti-Dsg3 autoantibodies were positive in 68 patients (85.0%) with mucocutaneous type, 10 patients (76.9%) with cutaneous type and 13 patients (86.7%) with mucous type.

The median value of anti-Dsg1 autoantibodies was 80 U/mL [0–1000] in PV patients and 182 U/mL [1–12381] in PF patients. The median value of anti-Dsg3 autoantibodies was 175 U/mL [0–8121] in patients with PV.

A level of anti-Dsg1 autoantibodies ≥ 100 U/mL at diagnosis was significantly associated with an increased risk for overall mortality among patients with PV (HR = 2.76 (95% CI, 1.01–7.5), p = 0.047), but not PF (p > 0.05), in the initial univariate analysis. When adjusted by age, the serum levels of anti-Dsg1 autoantibodies did not reach statistical significance as a risk factor for overall mortality (HR = 1.98 (95% CI, 0.71 – 5.53), p = 0.195, for age adjusted as continuous variable and HR = 2.37 (95% CI, 0.87 – 6.46), p = 0.093, for age adjusted as dichotomous variable). Also, patients with PV and a level of anti-Dsg1 autoantibodies ≥ 100 U/mL showed a lower OS rate at 1, 3, 5 and 10 years, compared with patients with a level of anti-Dsg1 autoantibodies <100 U/mL (p = 0.039). Patients with PF and a level of anti-Dsg1 autoantibodies ≥ 100 U/mL have also shown a lower OS rate at 3, 5 and 10 years, compared with patients with a level of anti-Dsg1 autoantibodies < 100 (p > 0.05). In patients with PV we didn't find statistically significant differences with respect to levels of anti-Dsg1 autoantibodies, when the groups were divided by age, coronary heart disease and cardiac arrhythmia. The median and interquartile range for anti-Dsg1 autoantibodies were: age at onset ≥ 65 years 104 (5.33 - 160.6) U/mL versus < 65 years 62.05 (12.65 - 187.25) U/mL, p = 0.972; coronary heart disease present 53.8 (7.7 - 128) versus absent 81 (13.3 - 189), p = 0.580; cardiac arrhythmia present 69.95 (9.1 - 131.35) versus absent 80.25 (13.95 - 188.5), p = 0.444.

An initial serum level of anti-Dsg3 autoantibodies ≥ 100 U/mL could not be identified as risk factor for overall mortality among patients with PV (p = 0.314). We have not observed statistically significant differences between OS rates of patients with anti-Dsg3 autoantibodies ≥ 100 U/mL and anti-Dsg3 autoantibodies < 100 U/mL (p = 0.506).

For the multivariate analysis we started with a model that used all the variables assessed in the univariate analysis, excluding some due either to collinearity issues (i.e., cardiac arrhythmia) or to limited data availability in all patients (e.g., erythrocyte sedimentation rate and dose of steroids), and then we used a stepwise selection procedure to identify important predictors.

For the multivariate analysis, age was assessed both as a continuous and dichotomous variable. The identified independent risk factors for overall mortality in patients with PV using age as a continuous variable were: age of onset (years) (adjusted HR = 1.12 [1.06 - 1.19], p < 0.001) and presence of coronary heart disease at diagnosis (adjusted HR = 3.04 [1.07 - 8.67], p = 0.037). To further explore the relevance of autoantibody levels as risk factor, we manually entered in the previous model the “autoantibody” variable and found the HRs for: anti-Dsg1 autoantibodies ≥ 100 U/mL 2.73 [0.92 – 8.13], p = 0.072; age of onset (years) 1.11 [1.05 – 1.17], p < 0.001; and presence of coronary heart disease at diagnosis 4.21 [1.38 – 12.83], p = 0.011.

The identified independent risk factors for overall mortality among patients with PV using age as a dichotomous variable were: age of onset ≥ 65 years) (adjusted HR = 7.82 [2.71 – 22.59], p < 0.001) and presence of coronary heart disease at diagnosis (adjusted HR = 2.97 [1.03 – 8.58], p = 0.044). Likewise we further explored by manually entering in the model the anti-Dsg1 autoantibodies ≥ 100 U/mL, and found the HR for: anti-Dsg1 autoantibodies ≥ 100 U/mL 3.26 [1.13 – 9.40], p = 0.029; age of onset ≥ 65 years HR = 7.49 [2.61 – 21.54], p < 0.001, and presence of coronary heart disease at diagnosis (adjusted HR = 4.23 [1.40 – 12.84], p = 0.011). In patients with PF, a model containing only the variable age was selected by the stepwise procedure.

## Discussion

An age of onset ≥ 65 years, presence of coronary heart disease, cardiac arrhythmia and a level of anti-Dsg1 autoantibodies ≥ 100 U/mL at diagnosis were found to be related to overall mortality in patients with PV. An age of onset ≥ 65 years was found to be related to overall mortality among patients with PF.

A considerable proportion of “early” deaths within the first year after diagnosis has been observed in our group (9 out of 17 patients with PV and 2 out of 5 patients with PF). Based on the causes and age of death in this group of patients described in the Results section and also based on the fact that the diagnosis has been made relatively early after onset, as mentioned above, we could speculate that the important percentage of ''early deaths'' has been due rather to an older age of onset and to pemphigus-related complications, than to a delayed initiation of treatment.

The results of the present study confirmed that general prognostic factors for survival, including older age of onset and cardiovascular disease, are associated with death also in patients with pemphigus. In line with our data, previous studies suggested that an increased age of onset (ranging 60–75 years) represents a risk factor for survival in patients with PV [3,10,11,13].

Regarding the cardiovascular disease, a higher risk of death by this cause in pemphigus population, compared to general population has been previously reported by Huang et al. [3]. Still, the overall survival in pemphigus patients with cardiovascular comorbidities, versus pemphigus patients without cardiovascular comorbidities has not yet been addressed. In addition to the direct cause of death, there are a few possible explanations for the association of cardiac diseases with an increased risk of overall mortality, in patients with pemphigus.

Presence of a chronic inflammatory background may represent a link between pemphigus, cardiovascular comorbidities and a lower overall survival, due to the direct involvement of various cytokines, such as TNF alpha and IL-6. Both molecules have been shown to be involved in the pathogenesis of pemphigus [20,21], as well as atherosclerosis and coronary heart disease [22]. Although we live in the era of TNF-alpha inhibitors, the role of these agents in pemphigus therapy is still controversial, as only a limited efficacy has been proved [23-26]. Therefore, while TNF-alpha inhibitors were shown to reduce development of cardiovascular disease in patients with psoriasis [27], no evidence regarding such an effect is available in pemphigus.

Additionally, an increased inflammatory status in pemphigus could lead to an increased disease activity and, consequently, to a poor outcome. In our cohort, presence of an inflammatory background in PV patients, illustrated by an ESR ≥ 30 mm/h, was shown to be associated with a statistically significant lower overall survival at 1,3,5 and 10 years, compared to patients with an ESR < 30 mm/h (p = 0.039). Rzany et al. demonstrated similar findings among patients with bullous pemphigoid [17]. Still, this association has not been previously reported in patients with pemphigus and could, in our opinion, be explained by the two theories mentioned above, regarding the presence of a subsequently increased disease activity or an increased cardiovascular risk, due to the chronic inflammatory background.

An alternatively, but not most likely explanation would be the cross-reactivity hypothesis. Desmosomes are cell adhesion molecules, known to maintain the integrity of various organs subjected to mechanical stress, such as the skin or the heart [28]. It has been shown that patients from El –Bagre, Colombia, affected by a new variant of endemic pemphigus foliaceus, displayed autoantibodies against multiple cardiac epitopes [29]. Additionally, patients with pemphigus and atypical variants of myocardial infarction developed a high titre of anti-heart autoantibodies, that diminished after corticotherapy [30].

As a paradox, smoking, a very-well known risk factor for cardiovascular comorbidities, was proved to show a limited beneficial effect in pemphigus outcome, due to nicotine, an agonist for the acethylcoline receptors, which are known to play an important role in the pathogenesis of pemphigus [31,32]. In our study, smokers with PV and PF demonstrated a higher OS rate at 1, 3, 5 and 10 years, compared with non-smokers, albeit not reaching statistical significance (p > 0.05).

Smoking, was defined cross-sectionally, as ''smoking at diagnosis''. Our data related to smoking should be interpreted cautiously, because our knowledge on the effects of smoking on the disease severity of pemphigus is limited and is based on few observational retrospective studies and case reports [31,33-37]. Therefore, a causal link between smoking and a clinical improvement in pemphigus cannot be established. Based on the current hypotheses, the actual current smoking exposure, rather than the cumulative long-term nicotine dose, is likely relevant for the pemphigus disease activity. Moreover, patients with severe forms of pemphigus tend to cease smoking, in order to improve their survival. In addition, a dose-dependent effect of nicotine on the disease severity in pemphigus has largely not yet been addressed thorough enough. Therefore, in an initial approach we did not go beyond correlating the smoker/non-smoker status with the survival. Nevertheless, smoking cannot be suggested as therapy in patients with pemphigus. Future studies addressing this issue in-depth in patients and animal models of pemphigus should clarify the role of nicotine in pemphigus and provide a basis for developing therapies using alternative cholinergic agents or similar derivates.

A remarcable finding of the present study is related to the prognostic relevance of the level of pemphigus autoantibodies at the time of diagnosis. Patients with PV and a level of anti-Dsg1 autoantibodies ≥ 100 U/mL showed a lower OS rate at 1, 3, 5 and 10 years, compared with patients with a level of anti-Dsg1 autoantibodies < 100 U/mL (p = 0.039). Thus, a level of anti-Dsg1 autoantibodies ≥100 U/mL at diagnosis represented a risk factor for overall mortality in patients with PV (p = 0.047), in the initial univariate analysis, but not when ajusted by age as continuous or dichotomous variable (p > 0.05). Patients with PF and a level of anti-Dsg1 autoantibodies ≥100 U/mL have also shown a lower OS rate at 3, 5 and 10 years, compared with patients with a level of anti-Dsg1 autoantibodies < 100 (p > 0.05). In multivariate analysis, level of anti-Dsg1 autoantibodies represented an independent risk factor for overall mortality in PV when explored by adding the anti-Dsg1 autoantibodies ≥ 100 U/mL to the initial model adjusted by age as dichotomous (p = 0.029), but not when used as a continuous variable (p > 0.05). In patients with PV we didn't find statistically significant differences with respect to anti-Dsg1 autoantibodies levels when subdivided by age, coronary heart disease and cardiac arrhythmia.

Nevertheless, our novel findings do not exclude that autoantibody levels may represent an independent risk factor for survival. This hypothesis should be further investigated in future studies, using larger patient groups.

While previous studies have mainly focused on assessing the association between the level of autoantibodies to desmogleins and disease activity and their predictive value for relapse in pemphigus [7,38,39]*,* it remains largely unknown whether the levels of Dsg1- and Dsg3-specific autoantibodies also represent a risk factor for overall mortality in patients with PV. One can only speculate that an increased titre of anti-Dsg1 autoantibodies, which are present in the mucocutaneous subtype of PV, associate with a more severe disease and, subsequently, reduced overall survival. Indeed, it has been suggested that, when co-occuring with Dsg3-specific antibodies, high levels of autoantibodies against Dsg1 associate with cutaneous involvement, which may result in an increased risk of cutaneous infections [40], in addition to difficulties in alimentation and subsequent malnutrition, due to painful mucosal erosions [41].

Along the same lines, a correlation between increased levels of anti-Dsg1 autoantibodies and a higher disease activity has previously been reported in patients with pemphigus [38,39]. Titres of anti-Dsg1 autoantibodies were associated with disease severity, in the cutaneous and especially in the mucocutaneous form of PV [38]. Regarding the association between the level of anti-Dsg3 autoantibodies and disease severity, the results are controversial. While some studies have associated a higher level of anti-Dsg3 autoantibodies with a higher disease [7,8], a recent study found no association between levels of anti-Dsg3 autoantibodies and disease severity in none of the PV subtypes (mucous, cutaneous and mucocutaneous) [38].

In agreement with this hypothesis, patients with mucous and cutaneous PV subtype respond better to therapy, while patients with mucocutaneous involvement develop more severe forms of disease [42]. In our study, we showed that levels of anti-Dsg 1 autoantibodies provide a prognostic information. Patients with mucocutaneous involvement, which represented the majority of PV cohort (74.1%), have also demonstrated a lower overall survival rate (p > 0.05), compared with patients with mucous and cutaneous involvement. As described above, the severity of the mucocutaneous type in PV is associated with an increased titre of anti-Dsg1 autoantibodies. Our results suggest that an increased titre of anti-Dsg1 autoantibodies in patients with this PV subtype lead to an increase in disease severity, with a higher resistance to therapy and a lower overall survival rate.

A limitation of our study is related to a low number of observations for several subgroup analyses, which hamper detecting small differences between the subgroups (e.g., cutaneous/mucous/mucocutaneous involvement or ethnicity).

## Conclusions

In conclusion, in addition to common prognostic factors, including older age and cardiovascular comorbidities, our study signals the level of anti-desmoglein 1 autoantibodies as a candidate prediction factor for overall survival that should be reinforced in further studies.

While this is the first detailed study of risk factors for overall mortality among patients with pemphigus from Romania, the findings of this study are likely to be replicated in pemphigus patients from other geographic areas or of other genetic background. The newly identified factors have major implications for the stratification of patients and design of personalized therapeutic plans in the clinical setting. In addition, they should greatly facilitate further epidemiological studies in pemphigus.

## Abbreviations

DIF: Direct immunofluorescence microscopy
Dsg1: Desmoglein 1
Dsg3: Desmoglein 3
ELISA: Enzyme-linked immunosorbent assay
ESR: Erythrocyte sedimentation rate
PV: Pemphigus vulgaris
PF: Pemphigus foliaceus
OS: Overall survival rate
HR: Hazard ratio
